# Intent Prediction of Multi-axial Ankle Motion Using Limited EMG Signals

**DOI:** 10.3389/fbioe.2019.00335

**Published:** 2019-11-19

**Authors:** Unéné Gregory, Lei Ren

**Affiliations:** School of Mechanical, Aerospace and Civil Engineering, University of Manchester, Manchester, United Kingdom

**Keywords:** classification tree, electromyography (EMG), intent prediction, linear discriminant analysis (LDA), multi-axial motion, transtibial powered prostheses

## Abstract

**Background:** In this study, different intent prediction strategies were explored with the objective of determining the best approach to predicting continuous multi-axial user motion based solely on surface EMG (electromyography) data. These strategies were explored as the first step to better facilitating control of a multi-axis transtibial powered prosthesis.

**Methods:** Based on data acquired from gait experiments, different data sets, prediction approaches and classification algorithms were explored. The effect of varying EMG electrode positioning was also tested. EMG data measured from three lower leg muscles was the sole data type used for making intent predictions. The motions to be predicted were along both the sagittal plane (foot dorsiflexion and plantarflexion) and the frontal plane (foot eversion and inversion).

**Results:** The deviation of EMG data from its optimal pattern led to a decrease in prediction accuracy of up to 23%. However, using features that were calculated based on a participant's specific walking pattern limited this loss of prediction accuracy as a result of EMG electrode placement. A decoupled data set, one wherein the terrain type was accounted for beforehand, yielded the highest intent prediction accuracy of 77.2%.

**Conclusions:** The results of this study highlighted the challenges faced when using very limited EMG data to predict multi-axial ankle motion. They also indicated that approaches that are more user-centric by design could led to more accurate motion predictions, possibly enabling more intuitive control.

## Introduction

Great strides have been made in the field of lower limb prostheses, particularly in the past few decades. The continued research and development conducted toward transtibial powered prostheses has enabled users to achieve gait that is comparable to that of healthy able-bodied individuals, whilst reducing their energy cost during ambulation (Hitt et al., 2009; Herr and Grabowski, 2012; Cherelle et al., 2014; Zhu et al., 2014).

The development of powered prostheses has necessitated the implementation of control systems to ensure desired functionality of these prostheses. Most of the control strategies implemented to date have been hierarchical in nature (Lawson et al., 2013; Hargrove et al., 2014; Young et al., 2014b; Yuan et al., 2014; Spanias et al., 2018). These have consisted of a high level (decision) control system, which deciphers the type of motion a user wants to perform, and a low level (execution) controller that oversees the actuation of said motion by the prosthesis. The high level controllers largely fall into two categories: machine learning based control approaches and proportional control methods, frequently using myoelectric signals (Dawley et al., 2013; Wang et al., 2013; Huang et al., 2014).

The design basis of a prosthetic device is to be a replacement of the amputated limb. As such, control strategies that can effectively reproduce the movement of the biological limb when executing both cyclical and random motions should be the next step in replacing the neural connections that have been lost due to amputation. Unlike for upper limb prostheses, there has been slow-paced development and commercialization of EMG based control strategies for lower limb prostheses. However, some progress has been made to facilitate EMG based control approaches (Au et al., 2005; Oskoei and Hu, 2007; Ha et al., 2011; Hargrove et al., 2011). Studies have also been conducted on the use of EMG signals for walking mode classification (Huang et al., 2009; Hoover et al., 2013).

Due to the non-stationary nature of electromyography (EMG) signals, particularly during walking, first identifying a user's locomotion mode has made it easier to implement control of lower limb powered prostheses (Huang et al., 2009). Using EMG data to predict user intent has been more successful for transfemoral (above-knee) amputees compared to transtibial (below-knee) amputees. This is mainly attributed to the larger number of muscles, mostly in the thigh, which can be used when controlling transfemoral prostheses, their relatively larger size compared to lower leg muscles and said muscles not been as affected by the ground-foot interaction during walking, unlike the lower leg muscles which are used for transtibial prostheses (Huang et al., 2009; Hoover et al., 2013).

The objective of this study was to investigate the effectiveness of different approaches for user intent prediction of multi-axis ankle motion. The intent prediction had to account for ankle-foot motion along two degrees of motion. Thus, the explored approaches had to predict user intent along the plane of progression and also out of said plane.

## Methods

### Data Acquisition Experiment Protocol

A gait experiment was conducted to study the biomechanical strategies used by able-bodied individuals when walking over a fixed, uneven terrain. The experiment was approved by the University of Manchester Research Ethics Committee (UREC reference 16086). Six able-bodied individuals participated in the gait experiment. They were all male and had no musculoskeletal limitations. The average height and weight of the participant group were 1.7 m (±0.08) and 74.2 kg (±12.2), respectively.

**Figure 1:**
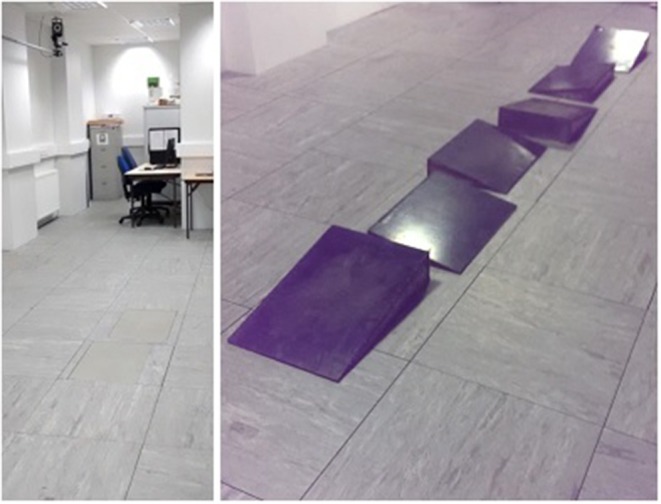
Level-Ground **(left)** and Uneven Terrain **(right)**.

Participants walked at three self-selected speeds, these were slow, normal, and fast. They walked over level-ground and a custom made fixed, uneven terrain shown in Figure 1. Walking over both terrain types made gait pattern comparison possible. Each participant completed 20 walking trials for each speed, over each type of terrain. This resulted in a total of 120 walking trials for each participant.

Kinematic and kinetic data were recorded for each participant using two 3D AMTI (Watertown, MA, USA) force plates and six Vicon (Oxford, UK) infrared cameras. The force plates were zeroed before conducting the uneven terrain trials to account for the weight of the introduced terrain. The addition of the uneven terrain had no effect on the calibration of the motion capture system. Surface EMG data was also recorded from eight muscles on each leg; namely the tibialis anterior (TA), medial and lateral gastrocnemius (MG and LG), rectus femoris (RF), medial and lateral vastus (VM and VL), biceps femoris (BF), and semitendinosus (SM). The EMG data was recorded using a Delsys Trigno wireless system (Natick, MA, USA). Even though EMG data from eight muscles was recorded, only data from the three lower leg muscles, namely TA, MG and LG, was used for intent prediction. This was done because the upper leg EMG data showed little variation as participants walked on the different terrains.

The three lower leg muscles used were chosen due to their relative size, proximity to the skin and their contribution to ankle-foot motion along both the frontal and sagittal plane. This made it easier and more effective to use surface EMG electrodes. It also minimized the likelihood of signal crosstalk, which tends to occur when measuring activation from muscles that are situated deeper in the body using surface electrodes. However, the shortfall of this approach was that key muscles that contribute more directly to ankle-foot motion along both the sagittal and frontal planes were omitted. These included the soleus, tibialis posterior, peroneus longus, brevis, and tertius muscles. However, as eversion is a combination of foot abduction and dorsiflexion and inversion is a combination of foot adduction and plantarflexion, the three muscles chosen formed a good basis from which to non-invasively explore intent prediction of multi-axial ankle motion.

The EMG data was automatically synchronized with data from the force plates and the motion capture system using the Vicon system. The kinematic and kinetic data were used to identify key phases of the gait cycle, including foot eversion and inversion as participants walked over the uneven terrain. Swing phase was identified based on the activation and deactivation of the two force plates in relation to each other, and also using the motion capture system. Identification of the gait cycle phases made it possible to segment the EMG data for both frontal and sagittal ankle-foot motion and use it to train the prediction approaches that were implemented.

The participants' muscle activation patterns and the magnitude of activation for the respective muscles were calculated from the measured EMG data (De Lisa, 1998). The EMG data was initially bandpass filtered at 20–450 Hz using a Butterworth filter to remove motion artifact and non-physiological signal content. It was then amplitude normalized based on each participant's maximum (isometric) voluntary contraction (MVC) (Yang and Winter, 1984; Halaki and Ginn, 2012). The normalized EMG data was then low pass filtered using a 2nd order recursive Butterworth filter with a cut-off frequency of 20 Hz to ensure motion artifacts were removed (De Luca, 1997).

### Feature Selection

The normalized EMG data was used in an incremental form with sequential analyses windows, rather than overlapping analyses windows. A window size of 150 ms was chosen based on previous studies (Huang et al., 2009, 2011; Varol et al., 2010) and our own trial-and-error process. Six features were chosen to be calculated from the EMG data. These were variance (VAR), waveform length (WL), integrated EMG (IEMG), 2nd order autoregression coefficients (AR), root mean square (RMS), and moving average (MAV). These features were chosen because they were time-domain features, meaning that their computation did not require signal transformation. Despite being a frequency domain feature, autoregression was chosen due to its relative ease of implementation and its reported classification accuracy (Huang et al., 2011; Young et al., 2013).

Five user motions were of interest, namely dorsiflexion, foot flat, plantarflexion, eversion, and inversion. Foot motion during swing phase of the gait cycle was omitted because said motion was not unique enough to be accurately classified based solely on EMG data. Omitting swing phase, and the prediction of this motion, also had no consequences in relation to controlling a prosthesis prototype as the foot was off the ground (non-load bearing) during this phase of the gait cycle. Additionally, swing phase foot motion could be reproduced using prosthesis mounted sensors without affecting the volitional nature of an implemented controller.

This study focused on predicting step-by-step user motion intent, unlike similar studies that predicted locomotion modes, such as level-ground walking, stair-ascent, ramp descent, etc. (Huang et al., 2011; Miller et al., 2013; Young et al., 2014a). This meant that for this study, a continuous data stream was received from the EMG sensors and motion prediction was perpetually performed at 150 ms intervals. As such, prediction was performed solely using EMG data and without mechanical sensor cues, “knowledge” of the walking environment or defining an environment mandated locomotion mode to be executed. This approach enabled more volitional control strategies to be implemented. The step-by-step intent prediction approach implemented could make it possible for transtibial powered prostheses users to perform non-cyclic motions in real-time.

Linear Discriminant Analysis (LDA) was used to determine which features, and which combinations thereof, yielded the most accurate prediction of user intent. LDA was chosen due to its ease of implementation, computational efficiency and classification accuracies which are comparable to more complex algorithms (Miller et al., 2013; Young et al., 2014a). Feature selection was done offline using EMG data acquired from the gait measurement described in section Data Acquisition Experiment Protocol. The gait experiment data set was segmented such that 70% of it was used for training while the remaining 30% was used for testing.

### Prediction Approaches

Three prediction approaches were explored. For all three approaches, features were calculated from the EMG data of the gait experiment discussed in section Data Acquisition Experiment Protocol. However, the data point of reference with regards to how the calculated features were used was different based on the prediction approach implemented. This meant that the features were implemented in varying ways specific to the different prediction approaches or calculated from different data subsets. The first approach used EMG features calculated from the aggregated data of the entire participant group to predict user intent. These features were not augmented in any way and were used as they were to make predictions for new participants. This was called the generic approach.

For the second approach, we first determined the degree to which a participant's walking style deviated from a defined optimum walking style. A participant's walking style was determined based on their lower leg muscle activation patterns during level ground gait. The optimum walking style was defined as a gait pattern wherein particular lower leg muscles activated at specific phases of the gait cycle (Winter, 1983; Winter and Yack, 1987). We then adjusted the participant's measured EMG data to better reflect the optimum walking style. This was done by biasing the necessary EMG channel(s). The features calculated from the aggregated data of the entire participant group were then used to make motion predictions for new participants. Thus, two additional steps were initially performed before using the features calculated from EMG data of the entire gait experiment participant group. This was called the biased approach.

For the third approach, six distinct walking styles were identified and labeled from the gait experiment data acquired as described in section Data Acquisition Experiment Protocol. The six walking styles were hyper TA, hyper MG, hyper LG, moderate MG, moderate LG, and an optimum walking. These were defined based on the degree to which they deviated from the optimum walking style. For this approach, new EMG features were calculated for each of the six walking styles identified. Intent prediction was then performed using these new features that were specific to a participant's walking style, which was one of the six identified. As such, this approach was more user-centric by design. It was aptly called the walking style approach. The differentiating factor for the three approaches was the EMG features they used to facilitate intent prediction.

### Classification Algorithms

In addition to using a LDA classifier, which was initially used for feature selection, three more classification algorithms were used to predict motion. As with exploring various prediction approaches, different classification algorithms were explored to determine which would yield the best accuracy for EMG based multi-axial motion prediction.

The first additional algorithm was a classification tree, a CART algorithm (Breiman et al., 1984). The second was a deterministic algorithm. Unlike the LDA and classification tree, it was not a machine learning based algorithm. Its classification was based on expectations of certain muscles being active at particular phases of the gait cycle, with respect to specific ankle-foot motions. The deterministic algorithm did not use EMG features to make predictions. Rather, a comparative approach using raw measured EMG data was implemented. The defined expectation of muscle activations with respect to gait phases is listed in Table 1. A mathematical description of this classifier is presented in (1).

(1) $$C_{\det}\to \{\begin{array}{c} 1,\;(p\wedge q)\\ \begin{array}{c} 3,\;(\sim p\wedge \sim q)\\ 4,\;(p\wedge \sim r)\wedge \sim q\\ 5,\;(q\wedge r)\wedge \sim p \end{array}\\ 2,\;otherwise \end{array}$$

where;

$$\begin{array}{l} p\to TA>MGas,q\to TA>LGas,and\;r\to MGas>LGas. \end{array}$$

**Table 1:** Muscle activation expectations of the deterministic algorithm.

**Movement type**	**Muscle activation**		
	**TA**	**MG**	**LG**
1. Dorsiflexion	Maximum	Minimum	
2. Foot flat	Maximum	Medium	
3. Plantarflexion	Minimum	Maximum	
4. Eversion	Maximum	Minimum	Medium
5. Inversion	Maximum	Medium	Minimum

Such that;

$$\begin{array}{l} \;TA=max\left(\;\sum_{i=1}^{n}TA\left(i\right)\;\right)\\ \;MGas=max\left(\;\sum_{i=1}^{n}MGas\left(i\right)\;\right)\\ LGas=max\left(\;\sum_{i=1}^{n}LGas\left(i\right)\;\right) \end{array}$$

**Figure 2:**
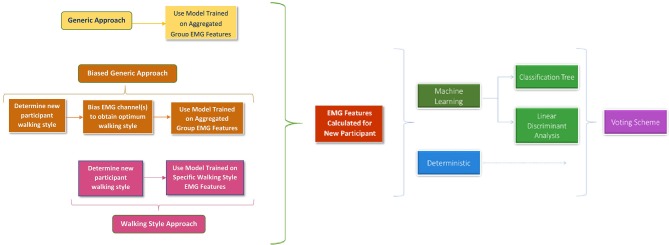
Graphical Representation of how the Prediction Approaches and Classification Algorithms Relate.

The third additional classification algorithm explored was a voting scheme. Its classification output was based on a majority agreement of the other three algorithms. If there was no agreement, the voting scheme output defaulted to that of the deterministic algorithm. A mathematical description of the voting scheme is presented (2). A graphical representation of how the four classification algorithms relate to each other and the three prediction approaches is presented in Figure 2.

(2) $$C_{VS}(t)=\{\begin{array}{c} \begin{array}{c} \begin{array}{c} C_{LDA}\left(t\right),C_{LDA} \end{array}(t)=C_{Tree}(t)\\ \begin{array}{c} C_{Tree}\left(t\right),C_{Tree} \end{array}(t)=C_{\det}(t) \end{array}\\ \begin{array}{c} C_{\det}\left(t\right),C_{\det} \end{array}(t)=C_{LDA}(t)\\ \begin{array}{c} C_{\det}\left(t\right),\;Otherwise \end{array} \end{array}$$

where;

*t* is the current prediction outcome.

*C*_*LDA*_, *C*_*Tree*_, and *C*_det_ are the prediction outputs of the LDA, classification tree and deterministic algorithm, respectively.

### Intent Prediction Experiment Protocol

A second gait experiment was conducted to assess the prediction accuracy of the different prediction approaches and classification algorithm combinations. Kinematic and EMG data were measured during the experiment. Three new participants (one female and two males) took part in the gait experiment. Their average height and weight were 1.74 m (±0.06) and 72.7 kg (±0.09), respectively. The participants walked at a self-selected normal speed. They walked on both level ground and the same fixed, uneven terrain used in the gait experiment of section Data Acquisition Experiment Protocol. Each participant completed eight walking trials over each type of terrain. This resulted in a total of 16 walking trials for each participant.

In order to evaluate the effect of EMG quality on the prediction accuracy, EMG data was collected for two electrode placement conditions. These were an optimal electrode (OE) and an electrode shift condition (ES). For the optimal electrode condition, the EMG electrodes were placed at their optimal anatomical locations. Though for the electrode shift condition, one of the three EMG electrodes was purposely placed 2 cm lower than its optimal location. The choice of which electrode to displace was based on the participant's walking style. For instance, the EMG electrode was shifted along the MG muscle for a participant that exhibited a hyper MG walking style.

The effect of using two different data sets for classifier training was also investigated. The first was a combined data set, it comprised of EMG data from both level ground and uneven terrain walking trials. The second data set was a decoupled data set, motion prediction was performed separately for level ground and uneven terrain walking.

The effect of using differing data sets was of interest because of the multi-axial nature of the motion being predicted. Determining which data set resulted in more accurate predictions would have implications on how and what kind of control strategy could be implemented for similar multi-axial transtibial powered prostheses.

### Statistical Analysis

Two-tailed paired *t*-tests were performed to investigate the effects of (1) EMG electrode positioning, (2) using different prediction approaches and classification algorithms, and (3) the data set, combined or decoupled, used for prediction. A confidence level of 95% was used for these analyses (α = 0.05).

## Results

### Feature Set Selection

Intent prediction was initially performed using a LDA classifier trained on the six individual EMG features and different combinations thereof from EMG data of the gait experiment described in section Data Acquisition Experiment Protocol. This was done to determine which feature combinations resulted in the highest classification accuracy. Three features that individually yielded the highest accuracies were IEMG (70%), WL (57%), and AR (57%).

However, combining all six features provided the highest prediction accuracy of 93%. The second-best performing feature combinations were VAR + IEMG + MAV and VAR + IEMG + AR + MAV which both reached accuracies of 80%. Therefore, all six features were used to evaluate the prediction accuracy of the different prediction approaches and classification algorithms.

### Combined Data Set

**Figure 3:**
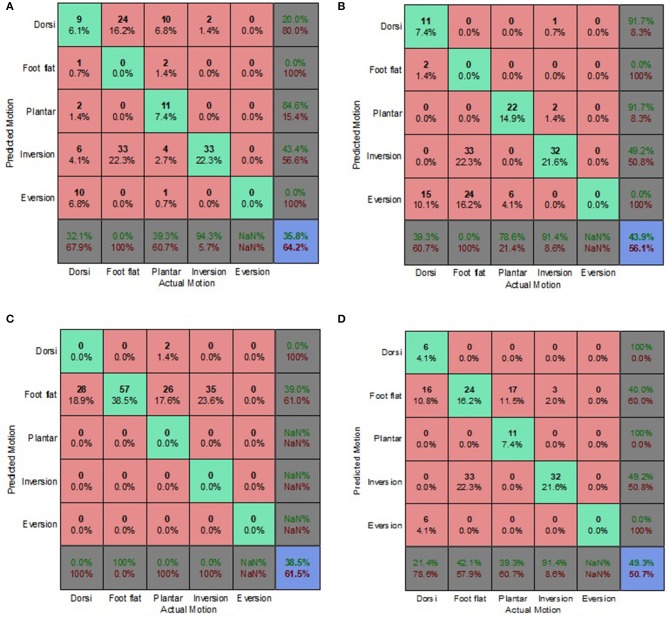
Optimal Condition Combined Data Set—Generic Approach. **(A)** Tree, **(B)** LDA, **(C)** Deterministic, and **(D)** Voting scheme.

**Figure 4:**
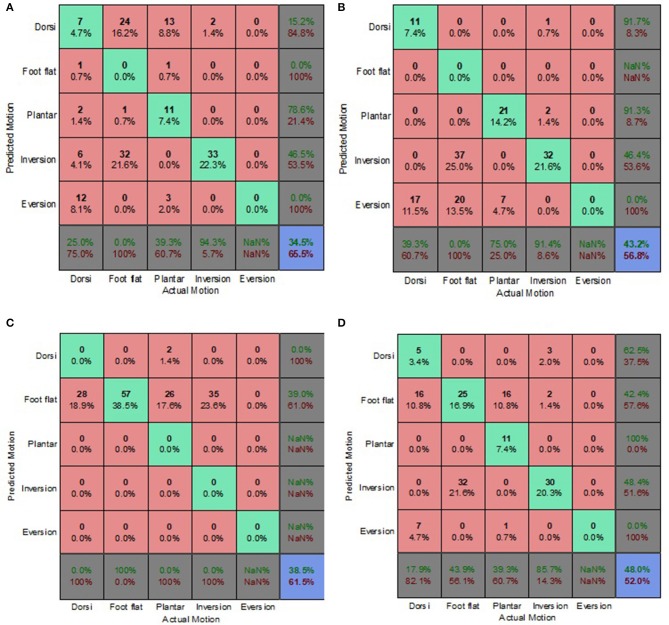
Optimal Condition Combined Data Set—Biased Generic Approach. **(A)** Tree, **(B)** LDA, **(C)** Deterministic, and **(D)** Voting scheme.

**Figure 5:**
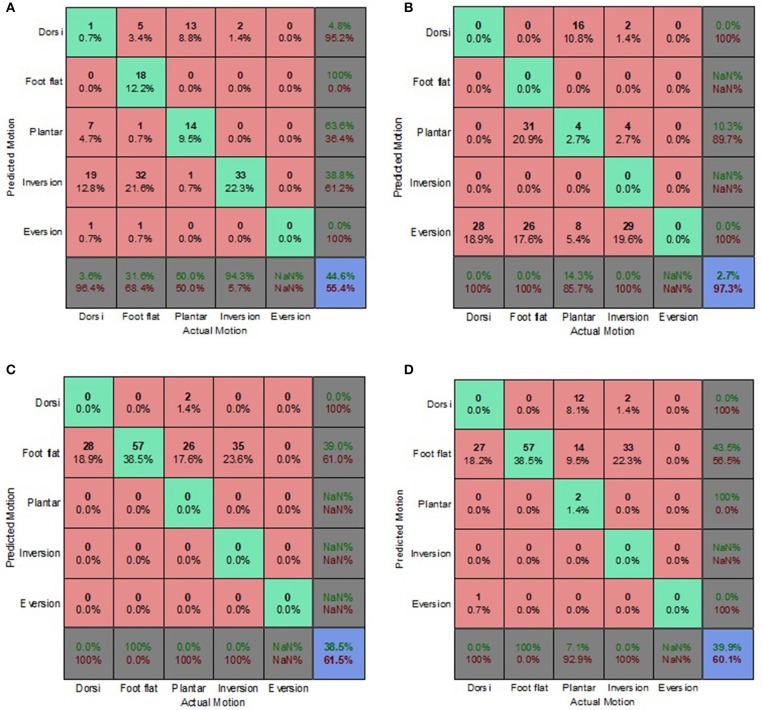
Optimal Condition Combined Data Set—Walking Style Approach. **(A)** Tree, **(B)** LDA, **(C)** Deterministic, and **(D)** Voting scheme.

**Figure 6:**
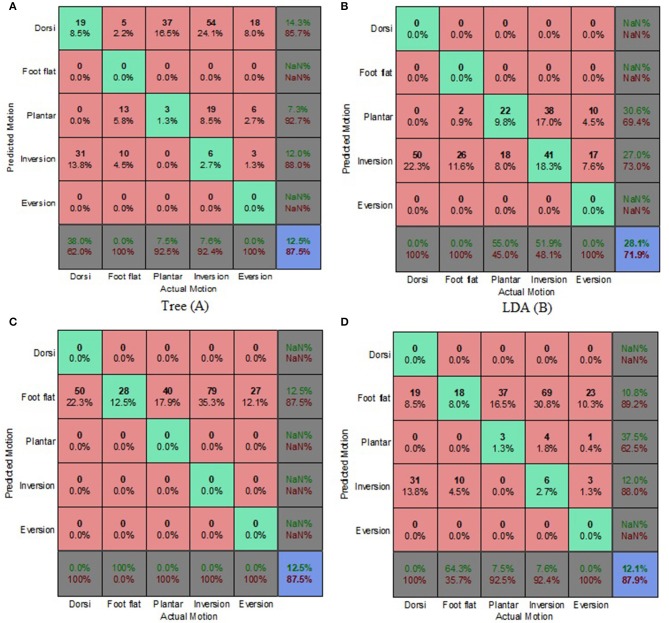
Electrode Shift Condition Combined Data Set—Generic Approach. **(A)** Tree, **(B)** LDA, **(C)** Deterministic, and **(D)** Voting scheme.

**Figure 7:**
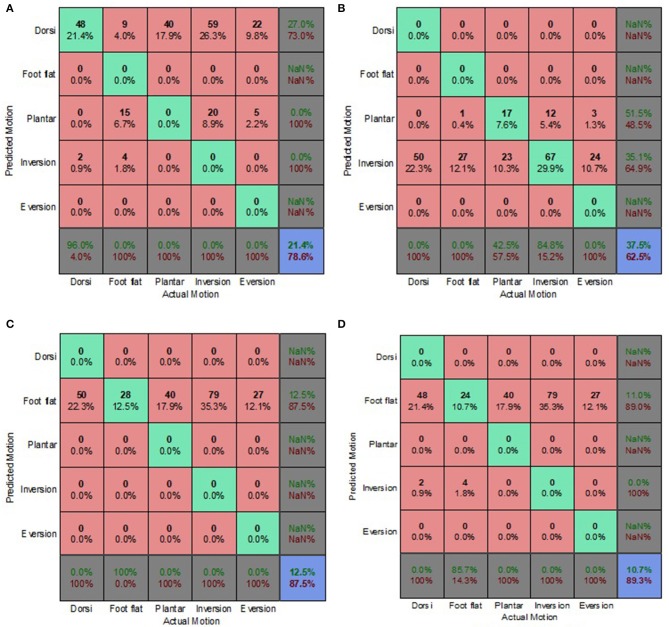
Electrode Shift Condition Combined Data Set—Biased Generic Approach. **(A)** Tree, **(B)** LDA, **(C)** Deterministic, and **(D)** Voting scheme.

**Figure 8:**
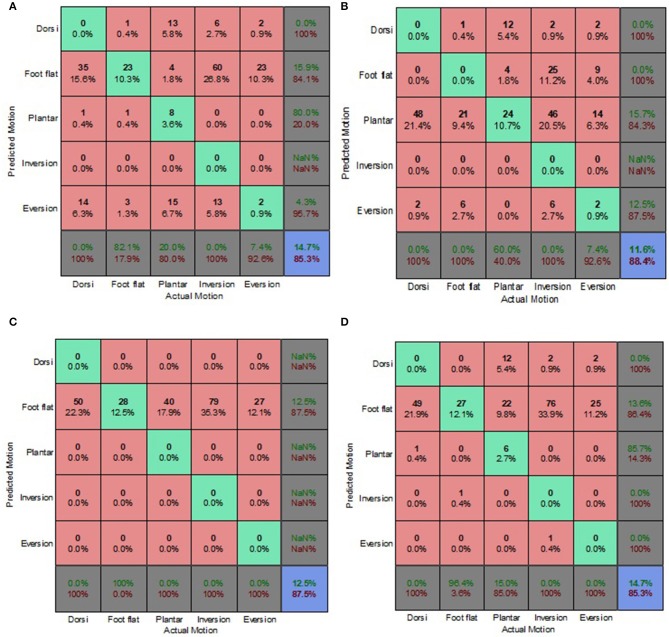
Electrode Shift Condition Combined Data Set—Walking Style Approach. **(A)** Tree, **(B)** LDA, **(C)** Deterministic, and **(D)** Voting scheme.

The prediction accuracy and misclassifications of each classification algorithm and prediction approach, for both electrode placement conditions are presented in Figures 3–**8** as confusion matrices. Most of the algorithms performed consistently better for the optimum electrode (OE) placement condition compared to the electrode shift (ES) condition. However, the prediction accuracies were significantly lower than those obtained during feature set selection (section Feature Set Selection).

The voting scheme algorithm produced the highest accuracy of 49.3% for the OE condition, indicating the benefits of aggregating multiple algorithms and playing to their individual strengths (Figure 3D). The deterministic algorithm achieved an accuracy of 38.5% for the OE condition (Figures 3C, 4C, 5C) and was unaffected by the implementation of different prediction approaches because it did not reply on EMG feature calculation. Its misclassifications were other motions being classified as foot flat.

The classification tree and LDA showed comparable accuracies for the generic and biased generic approaches, averaging 35 and 43%, respectively. However, the LDA performed poorly for the walking style approach (Figure 5B). The classification tree benefited from the implementation of the more user-centric walking style approach, achieving a prediction accuracy of 44.6% (Figure 5A). Overall for the OE condition, most misclassifications for both the classification tree and LDA were foot flat being misclassified as foot inversion, followed by foot flat being misclassified as foot eversion. With regards to the overall accuracy, the generic approach performed best for the OE condition.

On average, the prediction accuracies for the ES condition were 25.6, 20.6, and 18% lower than those of the OE condition for the generic, biased generic and walking style approaches, respectively (Figures 6–8). The machine learning based algorithms demonstrated their strengths of being better capable of handling imperfect input data. LDA performed best for the ES condition, indicating its robustness in the presence of flawed input data.

The biased generic approach was the best performing approach in terms of overall prediction accuracy for the ES condition (Figure 4). This suggested that a user-centric approach, such as the biased generic or walking style approach used in this study, could be beneficial when dealing with EMG signals that have deviated from their optimal state. LDA achieved the highest prediction accuracy of 37.5% for the ES condition.

Misclassifications for the deterministic algorithm were very similar for both the OE and ES conditions, with other motions being incorrectly classified as foot flat. The deterministic algorithm performed the worst for the ES condition, only achieving a prediction accuracy of 12.5%. The misclassifications of the classification tree, for the generic and biased generic approaches, suggested that predictions were slightly biased to dorsiflexion (Figures 6A, 7A). Whereas, for the walking style approach, the miscalculations of the algorithm changed to other motions being misclassified as foot flat (Figure 8A). For the LDA, most of the misclassifications were other motions being classified as foot inversion for the generic and biased generic approaches (Figures 6B, 7B), and other motions being misclassified as plantarflexion for the walking style approach (Figure 8B).

### Decoupled Data Set

**Figure 9:**
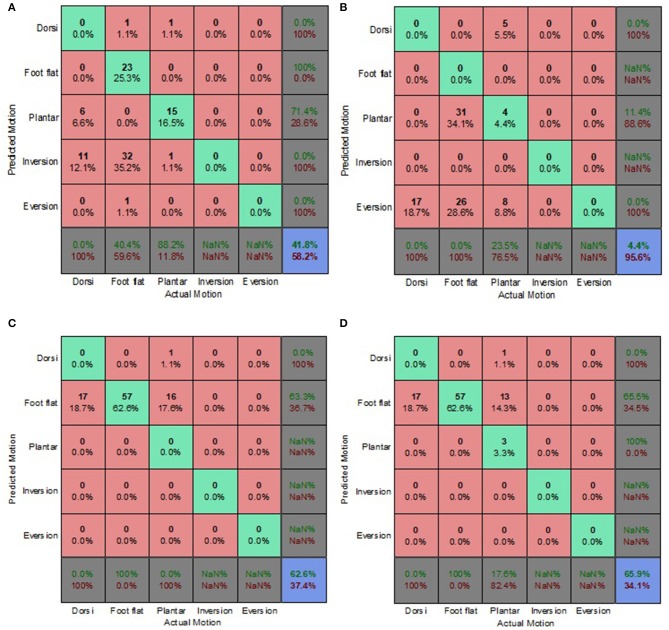
Optimal Condition Decoupled Data Set—LG Walking Style Approach. **(A)** Tree, **(B)** LDA, **(C)** Deterministic, and **(D)** Voting scheme.

**Figure 10:**
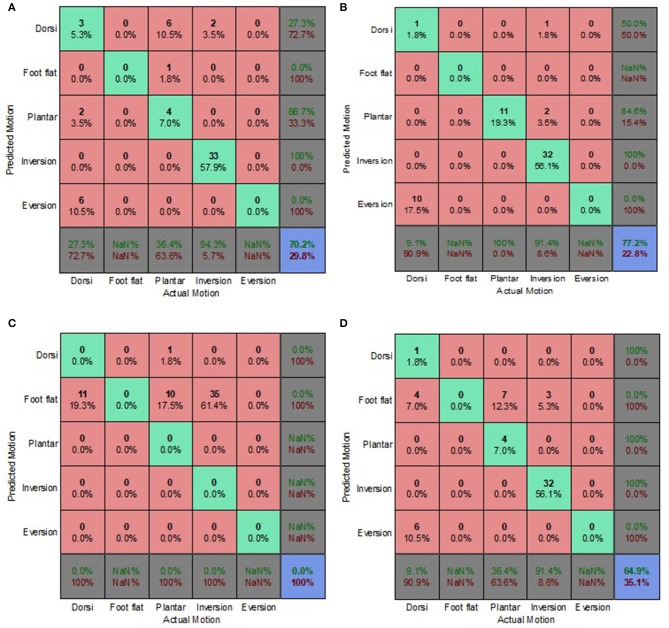
Optimal Condition Decoupled Data Set—UT Generic Approach. **(A)** Tree, **(B)** LDA, **(C)** Deterministic, and **(D)** Voting scheme.

**Figure 11:**
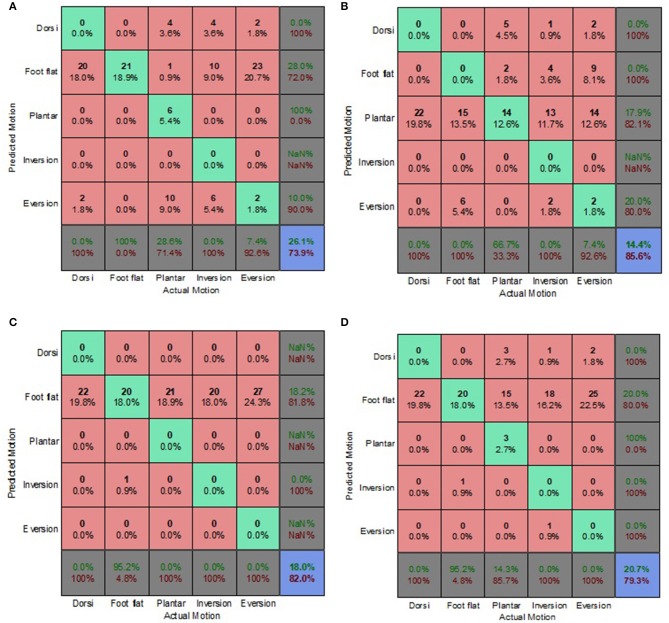
Electrode Shift Condition Decoupled Data Set—LG Walking Style Approach. **(A)** Tree, **(B)** LDA, **(C)** Deterministic, and **(D)** Voting scheme.

**Figure 12:**
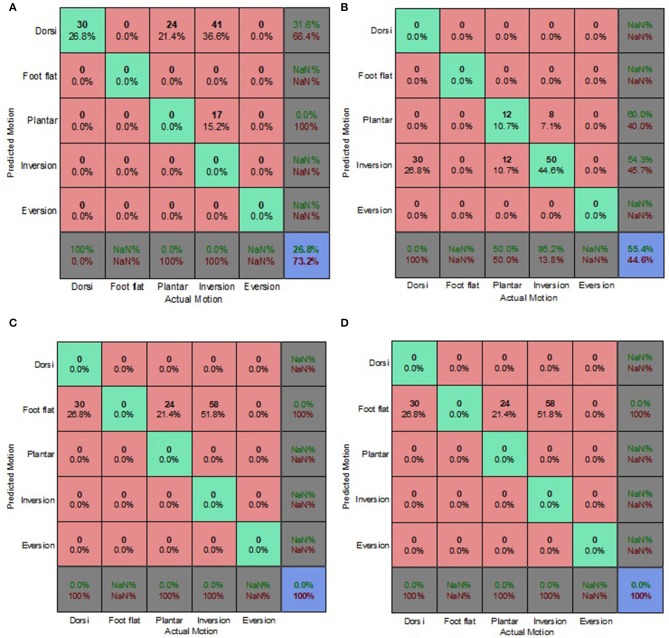
Electrode Shift Condition Decoupled Data Set—UT Biased Generic Approach. **(A)** Tree, **(B)** LDA, **(C)** Deterministic, and **(D)** Voting scheme.

Figures 9–12 present the prediction accuracies and algorithm misclassifications when intent predictions were made separately for the level ground and uneven terrain data. The machine learning based classification algorithms were retrained to classify the five ankle-foot motions detailed in section Feature Selection specifically for level ground and uneven terrain walking. Prediction accuracies for the optimal electrode (OE) condition were higher than those for the electrode shift (ES) condition.

Overall, the walking style approach was the best performing approach for the level ground data. The voting scheme algorithm achieved the highest prediction accuracy of 65.9% for the level ground OE condition. The deterministic algorithm fared better for the OE condition achieving a prediction accuracy of 62.6% (Figure 9C), compared to an 18% accuracy for the level ground ES condition (Figure 11C). The deterministic algorithm's errors of other motions being misclassified as foot flat would greatly hinder prosthesis user mobility.

Most of the algorithms misclassified other motions as foot flat for the level ground data set. The exceptions were the classification tree for the OE condition and the LDA for both electrode placement conditions. The classification tree misclassified other motions as foot inversion (Figure 9A). The LDA had difficulty differentiating between other motions and foot eversion for the level ground data set, which accounted for most of its misclassifications (Figure 9B). These misclassifications could have been attributed to the manner in which foot eversion data used for classifier training was initially obtained. The data were acquired during gait measurements which had participants performing maximum foot eversion whilst in a seated position with their heels in contact with the ground. This approach was similar to one taken by Au et al. during their study which involved a participant with an amputation (Au et al., 2008).

The classification tree achieved a prediction accuracy of 26.1% for the level ground ES condition using the walking style approach (Figure 11A), which was marginally better compared to its 21.4% accuracy for the combined data set using the biased generic approach (Figure 7A). This reinforced the hypothesis that a user-centric approach, such as the walking style approach used in this study, could be beneficial when dealing with EMG data that has deviated from its optimal pattern.

The LDA yielded the highest overall accuracy of 77.2%, this was for uneven terrain data using the OE condition (Figure 10B). This demonstrated the strength of the machine learning based algorithm to better distinguish between various patterns. When used with the biased generic approach, the LDA demonstrated its ability to better generalize to new and sub-optimum data, unlike the other algorithms, achieving a 55.4% accuracy (Figure 12B).

The deterministic algorithm performed dismally for the uneven terrain data, misclassifying all the motions. Its poor performance was due to the challenge of differentiating between foot eversion, foot flat and foot inversion based solely on raw EMG data. The voting scheme also performed poorly for the uneven terrain ES condition (Figure 12D). Interestingly, during the ES condition, the prediction accuracies for uneven terrain walking were generally higher than those for level ground walking. This was attributed to EMG patterns associated with performing foot motion along the frontal plane being more distinguishable than those of sagittal plane motion, particularly when using sub-optimal EMG data.

## Discussion

### EMG Electrode Placement

The effect of electrode placement was of interest due to the long-term focus of this study being to develop a control strategy for transtibial powered prostheses. As such, it is anticipated that electrode placement would not be entirely consistent during prosthesis use, even if low profile electrodes were embedded in the prosthesis socket. There has been more recent research conducted in developing transtibial prostheses with more than one degree of freedom (DoF). Examples of these include the Sparky 3 (Bellman et al., 2008) and a cable-driven 2 DoF ankle–foot prosthesis controlled using a microcontroller executing impedance control (Ficanha et al., 2016). To the author's knowledge, control strategies that have been implemented on 2DoF transtibial powered prostheses to date have used prostheses mounted sensors or data fusion approaches. These controllers have not solely used EMG data to drive the prostheses.

The highest overall prediction accuracies were achieved by the generic approach for the optimal electrode condition (Figure 10B) and the biased generic approach for the electrode shift condition (Figure 12B). There was a difference of 21.8% between the highest accuracies of these two electrode placement conditions. Statistical analysis indicated that EMG electrode placement (the quality of the EMG data) had a significant effect on the prediction accuracy (*p* < 0.05). Miller et al. reported similar findings for an electrode shift condition (Miller et al., 2013). Their study involved both able-bodied participants and those with unilateral transtibial amputations, all of whom retained control of their residual muscles. The reduction in classification accuracy as a result of electrode shift was observed for both participant groups. However, the largest decrease in accuracy of 60% was for an able-bodied participant, while the largest decrease in accuracy for the participant group with transtibial amputations was ~50%.

A deviation of the electrode position from its anatomically optimal placement, particularly when taking a participant's walking style into consideration, affected the quality of the measured EMG data used for motion prediction. It is worth noting that the electrode shift condition was only one representative cause of a reduction in EMG data quality in practical use. Other causes for a similar reduction in EMG data quality could occur due to muscle fatigue or signal noise introduced from external factors, such as excessive sweat.

### Prediction Approach vs. Classification Algorithm

The use of different prediction approaches did not have a significant effect on the prediction accuracies for both the combined data set (*p* = 0.6) and the decoupled data set (*p* = 0.62). Nevertheless, the poor performance of the walking style approach suggested that, in a broader sense, generalized data may have an advantage of minimizing nuances in individual walking patterns, particularly those leading to misclassifications of multi-axial motion. This was most evident when conducting motion predictions based on a combined data set wherein the worst overall performing prediction approach was the walking style.

Statistical analysis revealed that using different classifiers had a noticeable effect on the prediction accuracies. Though the effect thereof was marginal for the combined data set (*p* = 0.096) for both the OE and ES conditions. The different classifiers had no significant effect when investigating the OE condition for the decoupled data set (*p* = 0.79). Though a marginal effect was found when dealing with the ES condition for the decoupled data set (*p* = 0.07).

Among all the classification algorithms, the voting scheme yielded the highest overall prediction accuracy of 49.3% for the combined data set. This was followed by the classification tree at 44.6%. The performance of the voting scheme demonstrated the advantage of potentially compensating for deficiencies of individual classification algorithms and leveraging their specific strengths, particularly when classifying multi-faceted motions. The voting scheme stemmed from the observation that different classification algorithms performed differently when predicting certain types of motion. We also took a cue from previous studies that used a majority vote approach to make classifications (Varol et al., 2010; Huang et al., 2011; Young et al., 2014a). These studies used a classifier's time history, the past *n* decisions a classifier has made. A classification output triggered a change in locomotion mode only when a majority of the past sample windows gave the same output.

It is possible that with further development, the voting scheme could achieve improved prediction accuracy. However, its performance would depend on the individual classifiers used and on the manner in which the best classification output would be determined if a majority vote was not achieved. For instance, Huang et al. (2011) reported better classification accuracy using SVM over LDA, while Miller et al. (2013) reported that they observed similar accuracies for both LDA and SVM. This was also evident from the classification results; the voting scheme only had the highest classification accuracy 40% of the time. Hence, there would be a possibility of introducing unnecessary complexity with such an algorithm.

The performance of the deterministic algorithm suggested that machine learning based algorithms could be outperformed by simpler and less computationally intensive approaches. This also supported the implementation of EMG based proportional control approaches for powered prostheses that directly respond to user input, rather than first conducting motion prediction. Such approaches would not be susceptible to issues of generalization and robustness which often affect machine learning based approaches. This issue of controller generalization was also highlighted in a study by Young et al. (2013).

However, the performance of an algorithm not based on machine learning would depend on the type of data being used to make classifications. The disadvantage of using a deterministic algorithm is that its performance is likely to degrade in conditions wherein the input data is harder to segment and distinguish from other similar data, e.g., the foot flat vs. the foot eversion/inversion motion. Situations wherein there are many possible output groups could also affect the performance of such an algorithm. This was the case observed in this study. An example of this was a study by Miller et al. wherein they reported classifier misclassifications when distinguishing between different level ground walking speeds (Miller et al., 2013).

### Data Set vs. Prediction Accuracy

Overall, the statistical analysis showed that decoupling the data sets used for prediction had no significant effect on the prediction accuracies (*p* = 0.98). Nonetheless, higher prediction accuracies were achieved when using the decoupled data set for both the optimal electrode (OE) and electrode shift (ES) conditions. There were average increases in prediction accuracies of 10.3 and 3.5% for the OE and ES conditions, respectively, for the decoupled data set compared to the combined data set.

The prediction accuracies of the decoupled data set were more comparable to those reported in similar studies (Huang et al., 2011; Miller et al., 2013; Young et al., 2014a). Young et al. reported classification errors of ±28 and ±6% for transitional and steady-state data respectively, for level ground, ramp and stair traversal when using EMG data from nine muscles (Young et al., 2014a). The study presented in this paper used EMG data from only three lower leg muscles.

Unlike other studies that classified data from varying terrains which elicit distinct muscle activations, such as traversing stairs; this study classified EMG data from level ground and uneven terrain traversal. Walking over these two terrains resulted in minimal differences in the measured EMG data which, coupled with the limited number of EMG channels used, affected the resulting accuracy. Other studies highlighted the increase in misclassifications when using level ground data compared to data from traversing ramps or stairs (Miller et al., 2013; Young et al., 2014b).

The best performing combinations of prediction approach and classification algorithm are listed in Table 2. The performance of the generic and biased generic approaches indicated the benefit of using a greater range of training data when predicting multi-axial motion. This suggested that a greater pool of training data, with more variations, could lead to improved overall accuracy. The limitation of this study was the small participant group from which the measurement data was acquired. Using three EMG channels also affected the size of the feature set used for classification. Young et al. discussed the LDA's limitation in classifying non-stationary signals, such as lower limb EMG signals, but noted its ability to use a large feature set to classify new data having used a small sized data set for training (Young et al., 2014b).

**Table 2:** Best performing combinations of prediction approach and classification algorithm.

**Data set**	**Prediction approach**	**Classification algorithm**
	**Optimal electrode condition**	
Combined – LG + UT	Generic	Voting scheme
Decoupled – LG	Walking style	Voting scheme
Decoupled – UT	Generic	LDA
	**Electrode shift condition**	
Combined – LG + UT	Biased generic	LDA
Decoupled – LG	Walking style	Classification tree
Decoupled – UT	Biased generic	LDA

The performance of the deterministic algorithm for the OE condition, based on the level ground data set, suggested that a simple classification approach could be useful for multi-axial prosthetic control during every day, level ground walking. In the same light, the poor performance of the machine learning based classifiers highlighted one of the most challenging issues when developing control strategies for powered prostheses, data overfitting or classifier biasing. These issues could be overcome by using larger training data sets or by conducting more classifier training. However, these processes can be time consuming and may introduce more complexities to the approaches used.

The overall prediction results, particularly for the decoupled data set, suggested that higher accuracy and better performance could be achieved by using the intent prediction approaches presented in this study, especially if coupled with other sensors. These approaches took into consideration the type of walking terrain and the quality of the measured EMG data. This could lead to the development of an adaptive control approach that can adapt to the environment (walking terrain) and the user's conditions (EMG data quality) during use.

### Additional Considerations

This study only involved able-bodied participants. However, previous studies have reported that individuals with transtibial amputations tend to retain control of their residual muscles (Huang and Ferris, 2012; Seyedali et al., 2012; Silver-Thorn et al., 2012). In their study, Silver-Thorn et al. measured in-socket EMG data from three participants with transtibial amputations using low-profile surface electrodes (Silver-Thorn et al., 2012). They reported that the participants retained independent control of the residual muscles that were previously used to move the now amputated limb. However, the timing of said muscle activity was not always similar to that of able-bodied individuals.

The walking style approach would lend itself well to participants with transtibial amputations as the features used for intent prediction would be those based on their specific walking style. As such, differences in muscle activation timings or the repositioning of residual muscles following amputation would be accounted for. The biased generic approach could also be implemented as it accounts for a participant's walking style deviating from the defined optimum walking style.

On the other hand, new data would have to be measured from a large participant group with transtibial amputations to develop a generic approach for this group. Though given the unique muscle activations that individuals exhibit post amputation, this generic approach would still need to be “tuned” for each individual. This emphasizes the issue of controller generalization for prostheses and highlights the potential benefits of developing prediction approaches that are more user-centric by design, such as the biased generic and walking style approaches presented in this paper.

## Conclusions

The objective of this study was to explore the best combination of prediction approach and classification algorithm to predict multi-axial motion. This was done with the view of said strategy being the preceding step in facilitating control of a multi-axial transtibial powered prosthesis.

Gait experiments were conducted on both level ground and a fixed, uneven terrain to provide data sets for algorithm training and prediction accuracy assessment. A total of 12 combinations, comprising of four classification algorithms and three prediction approaches, were implemented and evaluated for both an optimal electrode and an electrode shift condition.

The results from this study revealed that a generic approach achieved higher overall prediction accuracy for the optimal electrode condition. Whereas, the biased generic and walking style approaches, which were more user-centric by design, performed better when the quality of EMG data used was negatively affected, as was the case for the electrode shift condition.

When assessing the performance of the classification algorithms using different data sets, it was found that the data set used had a greater effect on the prediction accuracy compared to the quality of the EMG data. This demonstrated that different prediction approaches performed best for specific data sets.

The importance of this study was in conducting a systematic investigation to determine the best way of predicting user intent for multi-axial ankle motion. This was done with the view of said prediction being used to drive and control a multi-axial transtibial powered prosthesis. The prediction accuracy achieved using a limited number of muscles was comparable to similar previous studies which used more muscles or a fusion of data from various types of sensors.

The results from this study suggested that more adaptive control strategies could enable more biologically similar motions for transtibial powered prostheses, including non-cyclic motions.

## Data Availability Statement

The datasets generated and/or analyzed during the current study are not publicly available due to pending commercialization pursuits but could be made available from the corresponding author on reasonable request.

## Ethics Statement

The study was approved by the University of Manchester Research Ethics Committee (UREC reference 16086) and all methods were carried out in accordance with the approved study protocol. The participants provided written informed consent before participation and consented to the publishing of their collected data.

## Author Contributions

UG developed the prediction approaches and the classifiers investigated. UG and LR designed the experiment. UG performed the experiment and analyzed the data. LR aided in the data interpretation. UG and LR prepared the manuscript and approved the final manuscript.

### Conflict of Interest

The authors declare that the research was conducted in the absence of any commercial or financial relationships that could be construed as a potential conflict of interest.

## References

[B1] Au S. Berniker M. Herr H. (2008). Powered ankle-foot prosthesis to assist level-ground and stair-descent gaits. J. Neural Netw. 21, 654–666. 10.1016/j.neunet.2008.03.006 18499394

[B2] Au S. K. Bonato P. Herr H. (2005). “An EMG-position controlled system for an active ankle-foot prosthesis: an initial experimental study,” in IEEE 9^*th*^ *ICORR* (Chicago, IL).

[B3] Bellman R. D. Holgate M. A. Sugar T. G. (2008). “SPARKy 3: design of an active robotic ankle prosthesis with two actuated degrees of freedom using regenerative kinetics,” in Proceedings of the 2nd Biennial IEEE/RAS-EMBS International Conference on Biomedical Robotics and Biomechatronics (Scottsdale, AZ). 10.1109/BIOROB.2008.4762887

[B4] Breiman L. Friedman J. H. Olshen R. A. Stone C. J. (1984). “Construction of trees from a learning sample,” in Classification and Regression Trees, 1st Edn. (New York, NY: Routledge), 297–317. 10.1201/9781315139470-11

[B5] Cherelle P. Grosu V. Matthys A. Vanderborght B. Lefeber D. (2014). Design and validation of the ankle mimicking prosthetic (AMP-) Foot 2.0. IEEE Trans. Neural Syst. Rehabil. Eng. 22, 138–148. 10.1109/TNSRE.2013.2282416 24122571

[B6] Dawley J. A. Fite K. B. Fulk G. D. (2013). “EMG control of a bionic knee prosthesis: exploiting muscle co-contractions for improved locomotor function,” in IEEE 13^*th*^ *ICORR* (Seattle, Washington). 10.1109/ICORR.2013.6650389 24187208

[B7] De Lisa J. A. (1998). Gait Analysis in the Science of Rehabilitation. D.o.V. Affairs, Editor, Baltimore Rehabilitation: US, 112.

[B8] De Luca C. J. (1997). The use of surface electromyography in biomechanics. J. Appl. Biomech. 13, 135–163. 10.1123/jab.13.2.135

[B9] Ficanha E. Rastgaar Aagaah M. Kaufman K. R. (2016). Cable-driven two degrees-of-freedom ankle–foot prosthesis. J. Med. Dev. 10, 030902–030902-2. 10.1115/1.4033734

[B10] Ha K. H. Varol H. A. Goldfarb M. (2011). Volitional control of a prosthetic knee using surface electromyography. IEEE Trans. Biomed. Eng. 58, 144–151. 10.1109/TBME.2010.2070840 20805047

[B11] Halaki M. Ginn K. (2012). “Normalization of EMG signals: to normalize or not to normalize and what to normalize to?,” in Computational Intelligence in Electromyography Analysis: A Perspective on Current Applications and Future Challenges, ed Naik G. R. (Scottsdale, AZ: InTech), 175–194. 10.5772/49957

[B12] Hargrove L. J. Simon A. M. Lipschutz R. D. Finucane S. B. Kuiken T. A. (2011). Real-time myoelectric control of knee and ankle motions for transfemoral amputees. JAMA 305, 1542–1544. 10.1001/jama.2011.465 21505133

[B13] Hargrove L. J. Simon A. M. Young A. Y. Lipschutz R. D. Finucane S. B. Smith D. (2014). Robotic leg control with EMG decoding in an amputee with nerve transfers. N. Engl. J. Med. 369, 1237–1242. 10.1056/NEJMoa1300126 24066744

[B14] Herr H. M. Grabowski A. M. (2012). Bionic ankle – foot prosthesis normalizes walking gait for persons with leg amputation. Proc. R. Soc. B Biol. Sci. 279, 457–464. 10.1098/rspb.2011.1194 21752817 PMC3234569

[B15] Hitt J. Sugar T. Holgate M. Bellman R. Hollander K. (2009). Robotic transtibial prosthesis with biomechanical energy regeneration. Indust. Robot Int. J. 36, 441–447. 10.1108/01439910910980169

[B16] Hoover C. D. Fulk G. D. Fite K. B. (2013). Stair ascent with a powered transfemoral prosthesis under direct myoelectric control. IEEE/ASME Trans. Mech. 18, 1191–1200. 10.1109/TMECH.2012.2200498

[B17] Huang H. Kuiken T. A. Lipschutz R. D. (2009). A strategy for identifying locomotion modes using surface electromyography. IEEE Trans. Biomed. Eng. 56, 65–73. 10.1109/TBME.2008.2003293 19224720 PMC3025288

[B18] Huang H. Zhang F. Hargrove L. J. Dou Z. Rogers D. R. Englehart K. B. (2011). Continuous locomotion-mode identification for prosthetic legs based on neuromuscular–mechanical fusion. IEEE Trans. Biomed. Eng. 58, 2867–2875. 10.1109/TBME.2011.2161671 21768042 PMC3235670

[B19] Huang S. Ferris D. P. (2012). Muscle activation patterns during walking from transtibial amputees recorded within the residual limb-prosthetic interface. J. Neuroeng. Rehabil. 9:55. 10.1186/1743-0003-9-55 22882763 PMC3582563

[B20] Huang S. Wensman J. P. Ferris D. P. (2014). An experimental powered lower limb prosthesis using proportional myoelectric control. J. Med. Dev. 8:2 10.1115/1.4026633

[B21] Lawson B. E. Varol H. A. Huff A. Erdemir E. Goldfarb M. (2013). Control of stair ascent and descent with a powered transfemoral prosthesis. IEEE Trans. Neural Syst. Rehabil. Eng. 21, 466–473. 10.1109/TNSRE.2012.2225640 23096120

[B22] Miller J. Beazer M. S. Hahn M. E. (2013). Myoelectric walking mode classification for transtibial amputees. IEEE Trans. Biomed. Eng. 60, 2745–2750. 10.1109/TBME.2013.2264466 23708765

[B23] Oskoei M. A. Hu H. (2007). Myoelectric control systems—A survey. Biomed. Signal Proces. Control 2, 275–294. 10.1016/j.bspc.2007.07.009

[B24] Seyedali M. Czerniecki J. M. Morgenroth D. C. Hahn M. E. (2012). Cocontraction patterns of trans-tibial amputee ankle and knee musculature during gait. J. Neuroeng. Rehabil. 9:29. 10.1186/1743-0003-9-29 22640660 PMC3480942

[B25] Silver-Thorn B. T. Current T. Kuhse B. (2012). Preliminary investigation of residual limb plantarflexion and dorsiflexion muscle activity during treadmill walking for trans-tibial amputees. Prosth. Ortho. Int. 36, 435–442. 10.1177/0309364612443379 22581661

[B26] Spanias J. A. Simon A. M. Finucane S. B. Perreault E. J. Hargrove L. J. (2018). Online adaptive neural control of a robotic lower limb prosthesis. J. Neural Eng. 15:016015. 10.1088/1741-2552/aa92a8 29019467 PMC5802866

[B27] Varol H. A. Sup F. Goldfarb M. (2010). Multiclass real-time intent recognition of a powered lower limb prosthesis. IEEE Trans. Biomed. Eng. 57, 542–551. 10.1109/TBME.2009.2034734 19846361 PMC2829115

[B28] Wang J. Kannape O. A. Herr H. M. (2013). “Proportional EMG control of ankle plantar flexion in a powered transtibial prosthesis,” in IEEE 13^*th*^ *ICORR* (Seattle, Washington). 10.1109/EMBC.2014.6943925 24187210

[B29] Winter D. A. (1983). Biomechanical motor patterns in normal walking. J. Motor Behav. 15, 302–330. 10.1080/00222895.1983.10735302 15151864

[B30] Winter D. A. Yack H. J. (1987). EMG profiles during normal human walking: Stride-to-stride and inter-subject variability. Electroencephalogr. Clin. Neurophysiol. 67, 402–411. 10.1016/0013-4694(87)90003-4 2444408

[B31] Yang J. F. Winter D. A. (1984). Electromyographic amplitude normalization methods: improving their sensitivity as diagnostic tools in gait analysis. Arch. Phys. Med. Rehabil. 65, 517–521. 6477083

[B32] Young A. J. Kuiken T. A. Hargrove L. J. (2014a). Analysis of using EMG and mechanical sensors to enhance intent recognition in powered lower limb prostheses. J. Neural Eng. 11:5. 10.1088/1741-2560/11/5/056021 25242111

[B33] Young A. J. Simon A. M. Fey N. P. Hargrove L. J. (2013). “Classifying the intent of novel users during human locomotion using powered lower limb prostheses,” in 6th International IEEE/EMBS Conference on Neural Engineering (NER) (San Diego, CA). 10.1109/NER.2013.6695934

[B34] Young A. J. Simon A. M. Hargrove L. J. (2014b). A training method for locomotion mode prediction using powered lower limb prostheses. IEEE Trans. Neural Syst. Rehabil. Eng. 22, 671–677. 10.1109/TNSRE.2013.2285101 24184753

[B35] Yuan K. Wang Q. Zhu J. Wang L. (2014). “Motion control of a robotic transtibial prosthesis during transitions between level ground and stairs,” in European Control Conference (ECC) (Strasbourg, France). 10.1109/ECC.2014.6862612

[B36] Zhu J. Wang Q. Wang L. (2014). On the design of a powered transtibial prosthesis with stiffness adaptable ankle and toe joints. IEEE Trans. Indust. Electron. 61, 4797–4807. 10.1109/TIE.2013.2293691

